# Zapnometinib treatment and influenza A virus infection modulate the HLA class I ligandome in human lung adenocarcinoma cells

**DOI:** 10.3389/fimmu.2026.1790267

**Published:** 2026-05-26

**Authors:** Hazem Hamza, Michael Ghosh, Hans-Georg Rammensee, Oliver Planz

**Affiliations:** 1 Institute of Immunology, University of Tübingen, Tübingen, Germany; 2 Virology Laboratory, Environmental Research Division, National Research Centre, Giza, Egypt; 3 Cluster of Excellence iFIT (EXC2180) “Image-Guided and Functionally Instructed Tumor Therapies”, University of Tübingen, Tübingen, Germany; 4 German Cancer Consortium (DKTK) and German Cancer Research Center (DKFZ), partner site Tübingen, Tübingen, Germany; 5 Cluster of Excellence CMFI (EXC2124) “Controlling Microbes to Fight Infections”, University of Tübingen, Tübingen, Germany

**Keywords:** gene ontology, HLA class I, influenza virus, ligandome, MEK inhibitors, zapnometinib

## Abstract

**Introduction:**

Influenza viruses continue to pose a global health threat, and available antiviral therapies are limited by resistance and reduced efficacy. Host-directed drugs such as MEK inhibitors have emerged as promising alternatives. Zapnometinib, a clinical-stage MEK inhibitor, has shown both antiviral and immunomodulatory activity. However, its impact on antigen presentation at the level of the HLA-I ligandome has not been investigated.

**Methods:**

Label-free LC–MS/MS was applied to analyze HLA-I–presented peptides in human lung adenocarcinoma Calu-3 cells. Cells were infected with Influenza A virus (IAV) H3N2/Fukui and treated with zapnometinib. Surface HLA-I expression was quantified by flow cytometry, and immunopeptidomic analyses were applied to assess ligandome alterations and functional pathway enrichment.

**Results:**

Zapnometinib treatment and IAV infection did not significantly alter HLA-I surface expression (fold changes 1.01-1.13, p > 0.05). Immunopeptidomics revealed allotype-specific changes in the relative abundance of HLA-I-presented peptides (approximately 3–12% change per allotype), and statistically significant modulation of defined ligand subsets (about 3–14% of ligands per condition; log_2_ fold change ≥ 2, adjusted p < 0.05). Functional annotation analysis showed condition-specific enrichment in distinct cellular pathways, such as interferon-induced pathways, including IFI16 upregulation during infection and its downregulation upon MEK inhibition, indicating selective modulation of antiviral responses, cell cycle and RNA-processing pathways under zapnometinib treatment, and adhesion-related pathways under combined treatment. Ligandome remodeling after IAV H3N2/Fukui infection strongly affected antiviral hubs (IFIH1, DHX58, IFI16), whereas zapnometinib caused no consistent or significant reduction.

**Discussion/conclusion:**

This study provides the first evidence that zapnometinib and IAV H3N2/Fukui induce remarkable effects on the HLA-I ligandome plasticity without substantially affecting overall HLA-I expression. These findings highlight the dual role of MEK inhibitors in modulating both viral replication and immune recognition through pathway- and allotype-specific changes in peptide presentation rather than bulk HLA-I levels. Our results support further investigation of zapnometinib as a host-directed antiviral strategy with the potential to contribute to targeted immunomodulation in antiviral therapy.

## 1 Introduction

Influenza virus and SARS-CoV-2 infections continue to pose major global public health challenges. While vaccination remains the most effective strategy for preventing the spread and emergence of new variants, potent antiviral drugs are critical for treating infections once disease has manifested. Currently, direct-acting antivirals (DAAs) such as neuraminidase inhibitors (e.g., Oseltamivir, Peramivir) are standard-of-care for influenza. However, the ongoing evolution and circulation of drug-resistant variants limit their long-term effectiveness (1–3), even against recently introduced agents such as Baloxavir Marboxil, a cap-dependent endonuclease inhibitor (4–9).

In contrast to DAAs, targeting host cellular factors provides an alternative strategy to combat viral infections and to mitigate resistance. Many RNA viruses exploit host signaling pathways that are essential for replication. Inhibition of these pathways, such as the mitogen-activated protein kinase (MAPK) cascade, can reduce the viral titers and modulate immune responses without inducing viral resistance (10–15). The Raf/MEK/ERK cascade, in particular, plays a critical role in influenza A virus (IAV) infection, where it enhances viral replication and progeny production by supporting viral RNA polymerase activity and viral ribonucleoprotein (vRNP) export (16–18). MEK inhibition disrupts these processes by retaining vRNP in the nucleus and thereby impeding viral replication (13–15, 19). Additionally, the nucleocapsid proteins of SARS-CoV can regulate COX-2 expression via PKCα signaling, which modulates the ERK/NF-κB cascade (20, 21).

As part of a drug repurposing approach, we previously evaluated the MEK inhibitor zapnometinib (ATR-002, formerly PD0184264). Zapnometinib, the active metabolite of CI-1040, targets MEK1 and MEK2 isoforms. Although originally developed as an antitumor drug, its oncological development was discontinued, despite having IC_50_ values comparable to its parent compound (22, 23). We later demonstrated broad antiviral efficacy of zapnometinib against multiple influenza A and B virus subtypes as well as SARS-CoV-2 (15, 19, 24, 25). In addition, zapnometinib acts synergistically when combined with standard-of-care DAAs (15, 26). Importantly, zapnometinib exhibits a dual mode of action: it interferes with viral replication through multiple mechanisms and modulates host immune responses by reducing cytokine and chemokine production. This has been demonstrated both *in vitro* and *in vivo*, including an LPS-induced acute lung injury mouse model (27, 28). Zapnometinib’s safety and tolerability were confirmed in a recent phase 1 clinical trial in healthy volunteers, which reported no major adverse effects. Furthermore, a phase 2 trial in hospitalized COVID-19 patients provided proof-of-concept for targeting the Raf/MEK/ERK pathway as a therapeutic approach in moderate to severe cases (29).

MAPK signaling pathways, particularly the Raf/MEK/ERK cascade, are central regulators of cell proliferation, differentiation, and survival (30, 31). These pathways also play a key role in modulating MHC expression in both normal and malignant cells (32–34). MEK inhibition alters surface expression of MHC class I molecules through both transcriptional and post-transcriptional regulation, thereby influencing antigen presentation to cytotoxic T lymphocytes (CTLs) (33, 35). Such changes can influence the immune system’s ability to recognize and eliminate cancer cells (36, 37). MEK pathway inhibition significantly affects peptide-MHC (pMHC) complex presentation on the cell surface, leading to quantitative and qualitative changes in the antigen presentation landscape. These alterations, shaped by the unique binding preferences of individual HLA class I allotypes, selectively reshape the composition of HLA class I allotype ligandomes and potentially influence T-cell recognition and immune responses (35, 38). Additionally, MEK inhibition can induce cellular stress responses and alter protein synthesis and degradation, leading to the presentation of novel peptides not usually displayed under normal conditions (39). These treatment-associated peptides may act as neoantigens, enhancing immune recognition and potentially improving the efficacy of immunotherapies (40).

In this study, we investigated whether zapnometinib significantly influences the HLA ligandome. We specifically examined its effects on HLA class I surface expression, HLA allotype distribution, and the potential presentation of novel peptides. By assessing these parameters, we aimed to clarify how MEK inhibition modulates the ligandome and to better understand its implications for antiviral and anticancer applications.

## 2 Materials and methods

### 2.1 Drug, cells, and virus

Zapnometinib (PD0184264) [2-(2-chloro-4-iodophenylamino)-N-3,4-difluorobenzoic acid] was synthesized at ChemCon GmbH (Freiburg, Germany). Human lung adenocarcinoma Calu-3 cells (ATCC^®^ HTB-55) were cultured in Minimum Essential Medium (MEM, Gibco, Thermo Fisher Scientific, Waltham, MA, USA, cat. no. 21430-079) supplemented with 10% fetal bovine serum (FBS, Capricorn Scientific GmbH, Dreihausen, Germany, cat. no. FBS-11A), 2 mM L-glutamine (Sigma-Aldrich, Merck KGaA, Germany; cat. no. G7513), 1 mM sodium pyruvate (Sigma-Aldrich, Merck KGaA, Darmstadt, Germany; cat. no. S8636), 1% non-essential amino acids (Sigma-Aldrich, Merck KGaA, Darmstadt, Germany; cat. no. M7145), and 1% penicillin/streptomycin (P/S, Sigma-Aldrich, Merck KGaA, Darmstadt, Germany; cat. no. P4333). HLA typing of Calu-3 cells (HLA-A*24:02, HLA-A*68:01, HLA-B*07:02, HLA-B*51:01, HLA-C*15:02) was obtained from the TRON Cell Line Portal (41). Madin-Darby canine kidney cells (MDCK II, ATCC^®^ CRL-2936™) were cultured in Iscove’s Modified Dulbecco’s Medium (IMDM, Gibco, Thermo Fisher Scientific, Waltham, MA, USA, cat. no. 12440053) and used to propagate IAV strain A/Fukui/20/2004 (H3N2/Fukui).

### 2.2 Infection of cells

Calu-3 cells were infected with IAV H3N2/Fukui at a multiplicity of infection (MOI) of 0.1. After 1 h, the inoculum was removed, cells were washed with Phosphate-Buffered Saline (PBS, Gibco, Thermo Fisher Scientific, Waltham, MA, USA, cat. no.14190) and overlaid with infection medium (DMEM supplemented with 0.3% bovine serum albumin (BSA, Carl Roth, Karlsruhe, Germany; cat. no. 0163.4), 1% P/S, and 1 µg/ml L-1-tosylamido-2-phenylethyl chloromethyl ketone (TPCK)-treated trypsin (Sigma-Aldrich, Merck KGaA, Darmstadt, Germany; cat. no. 4352157)). Zapnometinib was added at 50 µM, while 0.1% DMSO (Merck KGaA, Darmstadt, Germany, cat. no. 102952) served as the vehicle control. Uninfected cells were included as a mock control. Cells were harvested 24 h post-infection (hpi) using enzyme-free cell dissociation buffer (Gibco, Thermo Fisher Scientific, Waltham, MA, USA, cat. no. 13151014), washed twice with cold PBS, and stored at –80 °C.

### 2.3 Determination of HLA-I surface expression

HLA surface expression of Calu-3 cells was evaluated using QIFIKIT quantitative flow cytometric assay kit (Agilent Dako, Glostrup, Denmark, cat. no. K0078) according to the manufacturer’s instructions. This assay incorporates Calibration Beads for standard curve generation (log MFI vs. log ABC, R²>0.99) and Set-Up Beads for window-of-analysis, enabling detection of >10% HLA-I changes (CV<3%). In brief, both treated and infected Calu-3 cells were stained with the W6/32 monoclonal antibody (mAb) (pan-HLA class I specific for HLA-A, -B, and -C alleles, produced in-house) in triplicates or IgG isotype control (BioLegend, San Diego, CA, USA, cat. no. 400202), followed by secondary staining with FITC-conjugated rabbit-anti-mouse F(ab′)2 fragments (Agilent Dako, Glostrup, Denmark, cat. no. K0078) alongside QIFIKIT quantification beads (Agilent Dako, Glostrup, Denmark, cat. no. K0078). Untreated cells served as baseline for relative fold-change calculations. Data were analyzed using FlowJo software 10.8 (FlowJo LLC, BD Biosciences).

### 2.4 Immunoprecipitation of HLA-I-peptide complexes and peptide isolation

HLA class I molecules were isolated using a standard immunoaffinity purification procedure as previously described (42, 43). Briefly, frozen cells were lysed using 10 mM CHAPS (AppliChem, Darmstadt, Germany, cat. no. A1099) buffer prepared in PBS, containing a complete protease inhibitor (Roche, Mannheim, Germany, cat. no. 11697498001). The cell lysates were homogenized by applying pulsed sonification. Thereafter, the HLA-I molecules were purified from lysates by immunoaffinity chromatography with the pan-HLA class I specific W6/32 antibody coupled to CNBr-activated sepharose (GE Healthcare, Chicago, IL, USA, cat. no. 17043001). HLA-associated peptides were eluted with 0.2% TFA followed by ultrafiltration of the eluate using 3-kDa Amicon filter units (Merck Millipore, Burlington, MA, USA, cat. no. UFC5003). Desalting and concentration steps were accomplished by ZipTip C18 (Merck Millipore, Burlington, MA, USA, cat. no. ZTC18S096) and 0.1% TFA, and elution was performed with 32% Acetonitrile (AcN)/0.2% TFA. The final volume of the eluate was reduced by vacuum centrifugation.

### 2.5 Liquid chromatography–tandem mass spectrometry

Purified HLA ligands were separated by reverse-phase liquid chromatography (HPLC; UltiMate 3000 RSLCnano system, Dionex, Sunnyvale, CA, USA), using a 75 μm × 2 cm trapping column (Thermo Fisher Scientific, Waltham, MA, USA). Ligands separation was accomplished using a 50 μm × 25 cm separation column (Thermo Fisher Scientific) by applying a gradient ranging from 2.4 to 32.0% of acetonitrile for 90 min. For MS/MS, a top-speed collision-induced dissociation fragmentation method generating ion trap MS/MS spectra in the mass range 400–650 m/z was used with an online-coupled LTQ Orbitrap Fusion Lumos mass spectrometer (Thermo Fisher Scientific, Waltham, MA, USA).

### 2.6 Database search and spectral annotation of HLA ligandome

Data processing was carried out using the SequestHT algorithm in the proteome Discoverer software 1.4 (Thermo Fisher Scientific, Waltham, MA, USA) against concatenated-reviewed FASTA sequences of the human proteome and IAV proteome of the strain used in this study retrieved from the UniProt database. Precursor mass tolerance was set to 5 ppm and fragment mass 0.02 Da. Percolator-assisted false discovery rate (FDR) algorithm (44) was implemented at a target value of q ≤ 0.05 (5% FDR). Oxidized methionine residue was allowed as a dynamic modification, search engine rank =1, and peptide lengths were limited to 8–12 AA. Peptides were annotated to their respective HLA motifs using NetMHCpan 4.0 (45) with percentile rank or IC50 score below 2% and 500 nm respectively. For multiple possible annotations, the HLA showing the lowest rank/score was selected.

### 2.7 Label-free quantification of HLA ligands

To determine the abundance of presented IAV-derived ligands, the label-free quantification (LFQ)-method was used as previously described by Nelde, Kowalewski (46). Briefly, prior to the LC-MS/MS analysis, the total number of cells for each treatment was normalized and LC-MS/MS analysis was performed in five technical replicates for each sample. The relative quantification of HLA ligands was performed by calculating the area under the curve (AUC) of the corresponding precursor-extracted ion chromatograms using Proteome Discoverer 1.4 (Thermo Fisher Scientific, Waltham, MA, USA). To contend with the common LFQ and data-dependent acquisition (DDA) MS proteomics issues (47–49), the LFQ strategy was implemented by lowering FDR cut-offs and using matching between runs to reduce missing values in quantitation. High-quality peptide spectrum matches were filtered for 5% FDR and subsequently screened for binding affinity to the respective HLA molecules. Volcano plots were generated using in-house R script (ver. 3.2) depicting a pairwise comparison of the ratios of the mean areas of the individual peptides in the five LFQ-MS runs of each condition. Significant modulations were computed as an adjusted p-value <0.05 and a fold change of ≥ log2 2-fold change using a two-tailed t-test with Benjamini–Hochberg correction.

### 2.8 Software and statistical analysis

Proteome Discoverer 1.4 (Thermo Fisher Scientific, Waltham, MA, USA) software was used to analyze the MS/MS data. Functional annotations from up- and downregulated HLA-I ligands (log2 fold-change ≥ ± 2, adj. p<0.05) were carried out using the DAVID platform (50, 51). Standard Gene Ontology (GO) categories were analyzed: Biological Process (BP; dynamic cellular processes), Cellular Component (CC; subcellular localization), and Molecular Function (MF; biochemical activities) to provide comprehensive, orthogonal functional insights. Terms were filtered for Benjamini-Hochberg corrected p-value <0.05, fold-enrichment >1.3, and ≥3 proteins per term. Bubble plots visualized term significance (x-axis: log2 fold-enrichment; y-axis: enriched terms; bubble size: number of annotated proteins; color: corrected p-value), complemented by chord diagrams showing gene-pathway networks (50, 51). An In-house R script was used for volcano plots and analysis of relative HLA ligand abundances. Overlap analysis was performed using jvenn interactive tool (52). Alterations of the HLA-I surface expression were evaluated using Brown-Forsythe ANOVA with Dunnett’s T3 multiple comparisons correction. Statistical analysis was conducted using GraphPad Prism ver. 9.3 (GraphPad Software, San Diego, CA, USA) and p values <0.05 were considered statistically significant. Data visualization was performed using R (ver. 4.2) and GraphPad Prism. Where applicable, statistical details for each experiment are described in the corresponding figure legends.

## 3 Results

### 3.1 Zapnometinib does not alter HLA-I surface expression in Calu-3 cells

**Figure 1:**
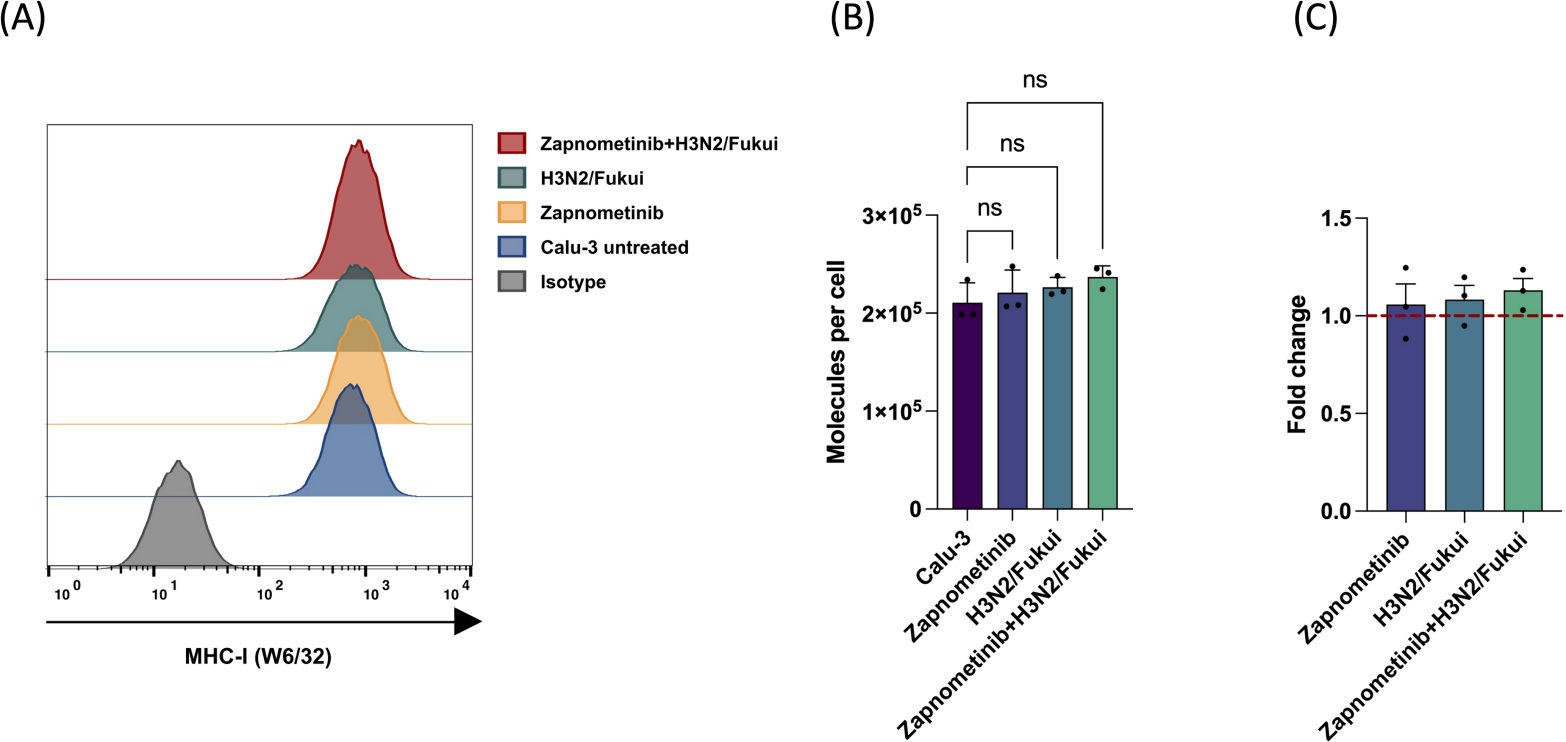
Effects of zapnometinib treatment and IAV infection on HLA-I surface expression in Calu-3 cells. Calu-3 cells were treated with zapnometinib and/or infected with IAV H3N2/Fukui, and HLA-I surface expression was analyzed using QIFIKIT-calibrated flow cytometry assay with the W6/32 monoclonal antibody (mAb) (pan-HLA class I specific for HLA-A, -B, and -C alleles). Untreated cells served as baseline for relative fold-change calculations. **(A)** Representative result of flow cytometry histograms showing HLA-I surface expression analysis (n=3). **(B)** Absolute quantification of HLA-I molecules on the surface of Calu-3 cells **(C)** Relative HLA-I surface expression normalized to untreated control (fold changes 1.01-1.13, Brown-Forsythe ANOVA with Dunnett’s T3 multiple comparisons test, all p>0.05). The statistical analysis was performed using Brown-Forsythe ANOVA with Dunnett’s T3 multiple comparisons correction. Flow cytometry data were analyzed using FlowJo software ver. 10.8 (FlowJo LLC, BD). Statistical analysis and visualization were performed using GraphPad Prism software ver. 9.3.

Previous studies have shown that anticancer agents, including MEK inhibitors, can induce notable changes in the HLA ligandome of cells, often accompanied by altered HLA class I surface expression (33, 35–37). To further explore these observations, we assessed the impact of zapnometinib on HLA-I surface expression in Calu-3 cells, both at baseline and following IAV infection. (**Figure 1A**). Our results showed that zapnometinib treatment did not significantly affect HLA-I expression (fold change 1.01 ± 0.1; **Figure 1C**, blue bar) compared with untreated cells, with mean expression levels of 220,888 versus 210,572 molecules/cell (**Figure 1B**). Consistent with previous reports that IAV and influenza B virus (IBV) modulate MHC-I profiles during late-stage infection (53, 54), we observed modest changes in IAV-infected cells (fold change 1.08 ± 0.07; **Figure 1C**, dark green bar). However, IAV-infected cells treated with zapnometinib showed comparable expression (fold change 1.13 ± 0.05; **Figure 1C**, light green bar), with mean values ranging from 226,518 to 237,036 molecules/cell (**Figures 1B, C**). Overall, these findings demonstrate that neither zapnometinib treatment nor IAV infection significantly alters HLA-I surface expression in Calu-3 cells (Brown–Forsythe ANOVA with Dunnett’s T3 multiple comparisons test, p > 0.05).

### 3.2 Zapnometinib treatment and IAV infection induce allotype-specific alterations in HLA-I peptide presentation in Calu-3 cells

**Figure 2:**
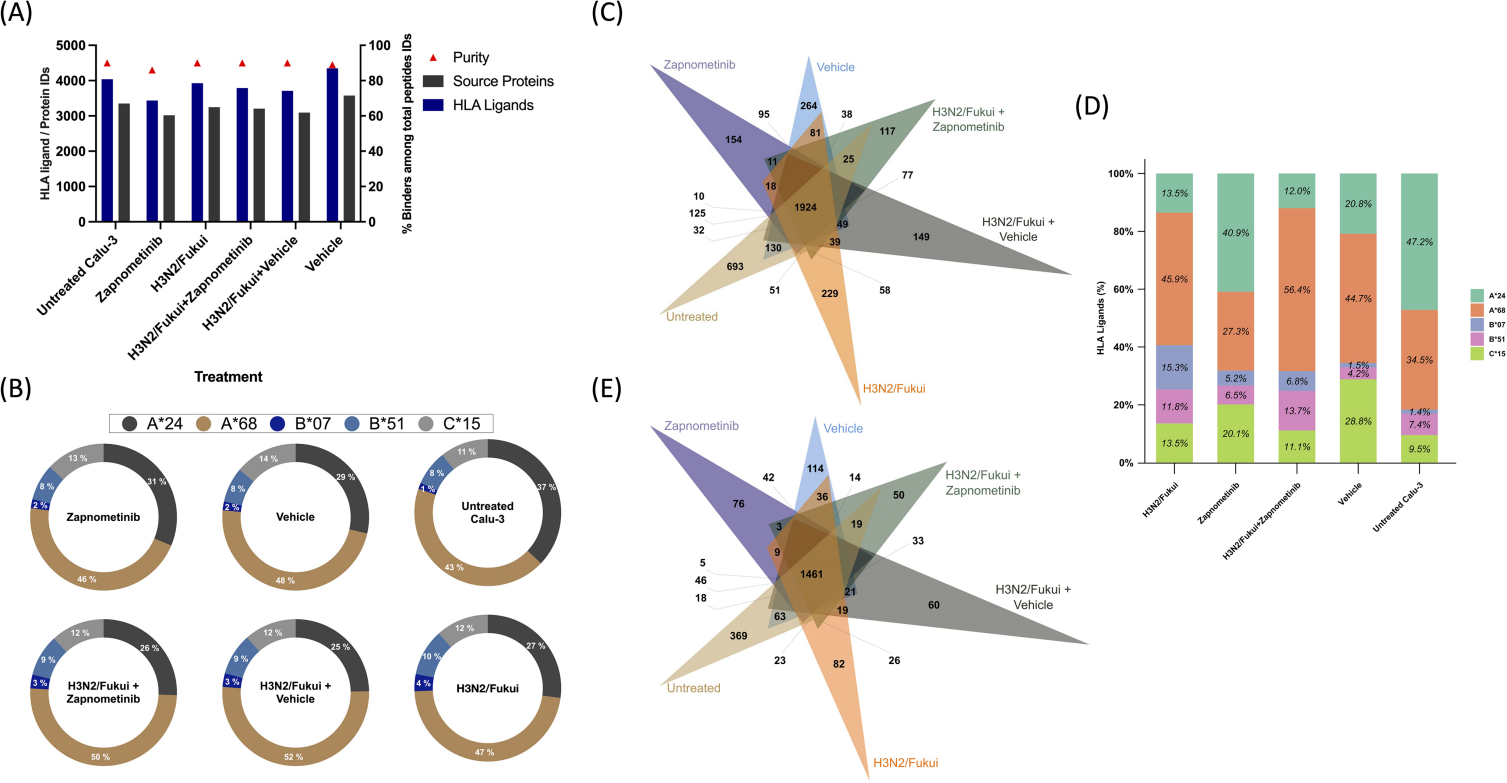
Mass spectrometric profiling of the Calu-3 cells HLA-I immunopeptidome following zapnometinib treatment and IAV infection. Calu-3 cells were treated with zapnometinib and/or infected with IAV H3N2/Fukui and HLA-I–bound peptides were isolated and analyzed by LC-MS/MS. Peptides were defined as HLA-I ligands if the NetMHCpan prediction showed percentile rank or IC50 score below ≤2% and 500 nM respectively. **(A)** Total number of HLA-I ligands (blue bars) and corresponding source proteins (black bars) identified under each condition. Red triangles indicate the purity of identified ligands (the percent of predicted ligands among the total identified peptides. **(B)** HLA-I allotype distribution of predicted ligands in zapnometinib-treated cells and IAV-infected cells. **(C)** Venn diagram showing overlap of HLA-I identified ligands between each treatment condition and mock control. **(D)** HLA-I allotype distribution of exclusively identified ligands under each treatment. **(E)** Venn diagram showing overlap of source proteins for HLA-I ligands respective for each treatment and infection conditions. A comprehensive list of identified peptides used for generating the figure can be found in the **Supplementary Material**. * Indicates statistical significance.

Mapping of HLA-I-presented ligands following zapnometinib treatment and/or IAV infection revealed a diverse repertoire ranging from 3,436 to 4,347 peptides per condition, representing 3,013–3,573 source proteins (**Figure 2A**). The lowest number of ligands was observed after zapnometinib treatment (3,436), compared with untreated Calu-3 cells (4,041) and vehicle controls (4,347). The purity of identified ligands, defined as the proportion of HLA ligands among total detected peptides, remained consistently high across conditions (86–90%). A comprehensive list of all peptides identified in this study can be found in the **Supplementary Material**.

To assess the impact of zapnometinib and IAV infection on HLA allotype-specific presentation, we compared the frequencies of allotype restrictions among naturally presented ligands (**Figure 2B**). Zapnometinib treatment reduced the proportion of ligands annotated to HLA-A24:02 by 6%, an effect further observed in IAV H3N2/Fukui-infected cells (10–12% decrease), with or without zapnometinib. In contrast, HLA-A68:01-restricted ligands increased by 3% after zapnometinib treatment and by 4–7% in IAV-infected cells, including those treated with zapnometinib. Minor alteration (1–3%) were also detected in HLA-B07:02, HLA-B51:01, and HLA-C*15:02 frequencies following IAV H3N2/Fukui infection and zapnometinib exposure relative to untreated controls. These allotype-specific frequency shifts were reproducible across five LFQ runs per condition and exceeded the technical variability of the assay (coefficient of variation <10%). Collectively, these results demonstrate that both IAV infection and zapnometinib treatment shape the HLA-I ligandome in an allotype-specific manner.

### 3.3 Modulation of HLA-I-presented peptides by zapnometinib treatment and IAV infection

To address common challenges in LFQ- and DDA-based MS proteomics, we employed an LFQ workflow with optimized FDR cutoffs and run-to-run matching to reduce missing values and improve quantitative accuracy of ligand identification across experimental conditions. Applying stringent criteria, involving high-quality peptide spectrum matches, predicted HLA-binding affinity, and a 5% FDR threshold, we conducted a preliminary qualitative comparison of HLA-I peptidomes in Calu-3 cells following zapnometinib treatment and/or IAV infection relative to untreated controls. Overlap analysis revealed substantial diversity in the HLA-I ligandome (**Figure 2C**). Zapnometinib treatment resulted in 4.5% unique peptides, whereas combined zapnometinib and IAV H3N2/Fukui infection yielded 3.1% unique peptides. Importantly, a novel IAV-derived epitope, HA_507–517_ (VYRDEALNNRF) from haemagglutinin, restricted to HLA-A*24:02, was identified exclusively in infected cells. This peptide was absent following zapnometinib treatment alone but proved immunogenic in HLA-matched healthy blood donors (54).

HLA allotype distribution among uniquely identified peptides showed that HLA-A68:01 as the prominent allotype in IAV H3N2/Fukui-infected cells (45.9%) and in infected cells treated with zapnometinib (56.4%), while HLA-A24:02 was most prominent in zapnometinib-only treated cells (**Figure 2D**). Comparative mapping of HLA-I ligand source proteins further underscored condition-specific repertoires. In zapnometinib-treated cells, 76 source proteins (3.5% of the mapped source proteome) were uniquely represented (**Figure 2E**). IAV H3N2/Fukui infection alone yielded 82 unique source proteins (3.4%), while infected cells treated with zapnometinib revealed 51 unique source proteins (2.1%) (**Figure 2E**).

### 3.4 HLA-I ligandome plasticity and functional annotation analysis following viral infection and zapnometinib treatment

**Figure 3:**
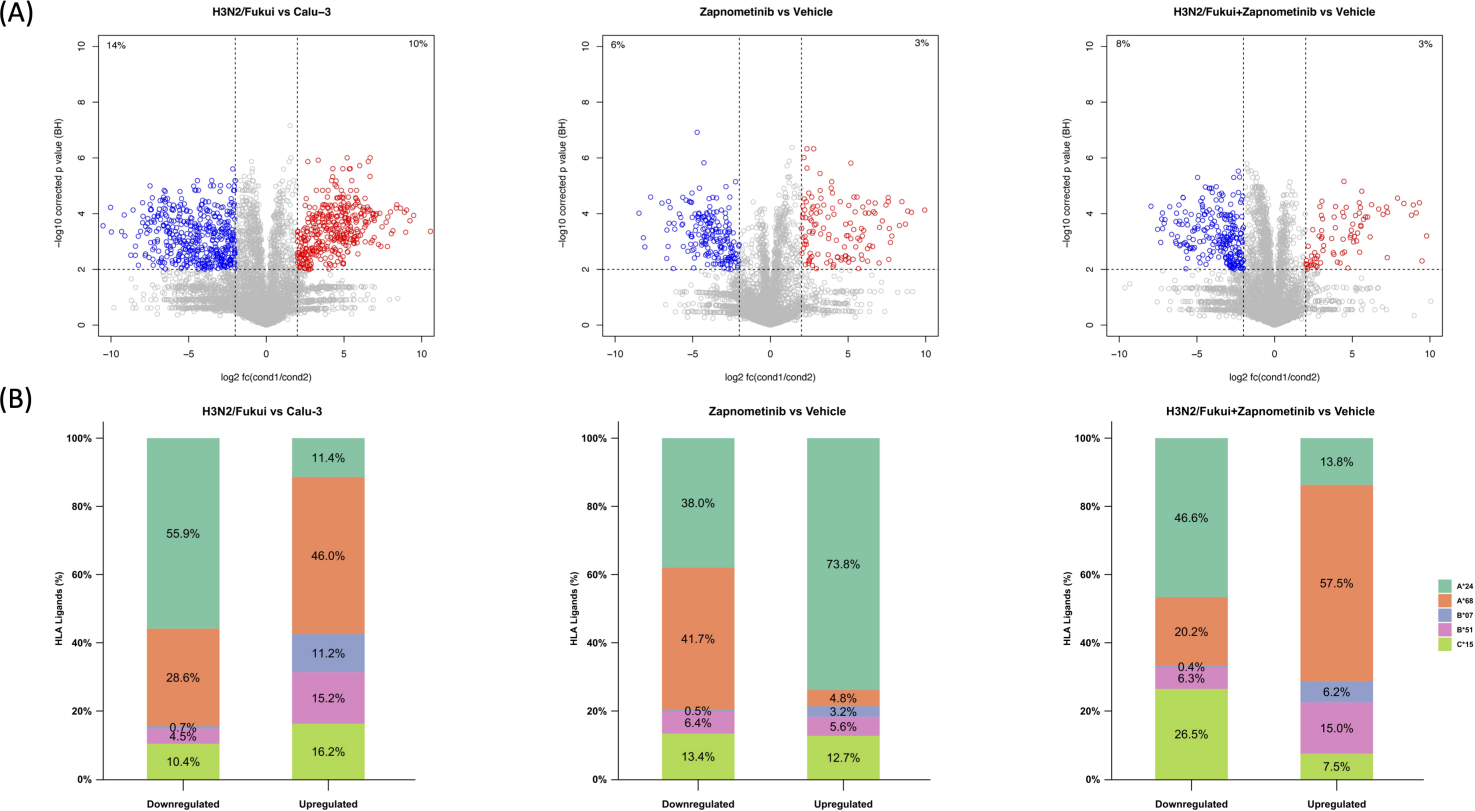
Qualitative and quantitative influence of zapnometinib treatment and IAV infection on the Calu-3 HLA-I peptidome. Calu-3 cells were treated with zapnometinib and/or infected with IAV H3N2/Fukui and HLA-I–bound peptides were isolated for LC-MS/MS analysis. **(A)** Volcano plots depicting the modulation and relative abundance of HLA-I identified ligands between the indicated conditions. Each point corresponds to an HLA-I ligand, the x-axis shows log2 fold change in relative abundance, and the y-axis indicates -log10 Benjamini–Hochberg corrected p-value. upregulated ligands (≥ 2-fold increase, adjusted *p*-value <0.05) are indicated in red and those significantly downregulated (≤-2-fold decrease, adjusted p < 0.05) are in blue. Percentages of significantly modulated ligands are indicated in the respective quadrants. **(B)** Distribution of HLA-I allotypes among the up- and downmodulated ligands. Data processing and visualization were performed in R (ver. 4.2) using in-house R scripts. A comprehensive list of identified peptides used for generating the figure can be found in the **Supplementary Material**.

Using LFQ-based quantitation, we evaluated the abundance of HLA-I ligands during IAV H3N2/Fukui infection and zapnometinib treatment. Substantial plasticity of the HLA-I ligandome was observed following IAV H3N2/Fukui infection, with 10% of ligands upregulated and 14% downregulated relative to untreated Calu-3 cells (**Figures 3A**, left) (meeting log2 fold-change ≥ 2 and Benjamini–Hochberg-adjusted p < 0.05 thresholds). Comparisons with vehicle controls confirmed that zapnometinib treatment alone (**Figures 3A**, middle), as well as combined treatment with IAV H3N2/Fukui infection, displayed similar patterns, with 3% of ligands upregulated and 6–8% downregulated (**Figures 3A**, right).

Analysis of HLA restriction patterns among modulated ligands revealed allotype-specific differences. In IAV H3N2/Fukui-infected cells, downregulated ligands were strongly enriched for HLA-A24:02 (55.9%), compared with only 11.4% representation in the upregulated fraction (**Figures 3B**, left). Conversely, HLA-A68:01 accounted for the largest proportion of upregulated ligands (46%), while HLA-B07:02 and HLA-B51:01 contributed 11.2–15.2%. Under zapnometinib treatment relative to vehicle, upregulated ligands were predominantly restricted by HLA-A24:02 (73.8%), whereas downregulated ligands were enriched for HLA-A68:01 (41.7%) (**Figures 3B**, middle). Infected cells treated with zapnometinib showed distribution patterns largely similar to IAV H3N2/Fukui infection alone, with the notable exception of HLA-C15:02, which represented 26.5% of the downregulated ligands, second only to HLA-A24:02 (46.6%) (**Figures 3B**, right). Together, these data indicate that statistically significant, but quantitatively restricted, subsets of the ligandome undergo allotype-specific remodeling under infection and MEK inhibition.

### 3.5 Functional annotation of HLA-I ligandome following viral infection and zapnometinib treatment

#### 3.5.1 Gene ontology enrichment analysis reveals distinct phenotypes across conditions

To assess how zapnometinib treatment alters host cell processes at the HLA-I–presented peptide level, Gene Ontology (GO) enrichment analysis of source proteins corresponding to up- and downregulated HLA-I ligands was performed using DAVID (50, 51). Three principal GO categories were analyzed: Biological Process (BP) for functional pathways, Cellular Component (CC) for subcellular localization, and Molecular Function (MF) for biochemical activities. This visualization approach allowed direct comparison of functional enrichment across IAV H3N2/Fukui infection, zapnometinib treatment, and their combination.

**Figure 4:**
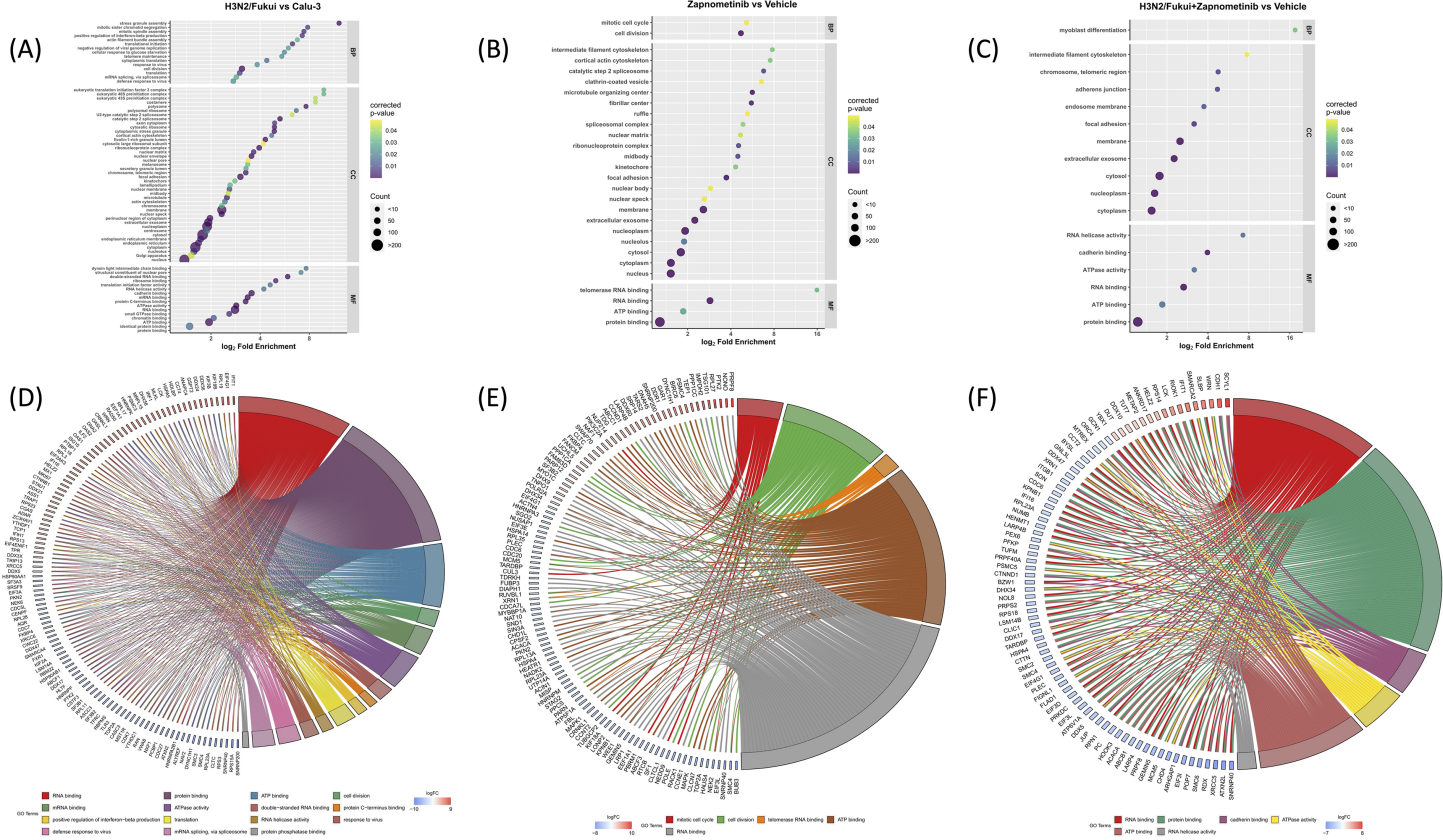
Gene Ontology (GO) analysis of MHC-I modulated ligands. **(A–C)** Bubble plot showing the Functional annotation analysis of enriched GO terms (CC, BP, or MF). Bubbles represent enriched terms; bubble size indicates the number of proteins annotated to that term, and bubble color denotes the enrichment statistical significance of Benjamini–Hochberg corrected *p*-value. The x-axis shows log2 fold-enrichment and the enriched terms are shown on the y-axis. The plots show only the GO terms above the cutoff criteria (fold enrichment >1.3, adjusted *p*-value <0.05, and ≥ 3 proteins per term). **(D–F)** Chord plots show the correlation between modulated HLA-I ligand source proteins and their enriched GO terms. The rectangles color scale following gene symbols represents the log2FC and arc colors indicate GO terms that match the legend. The plots show only GO terms that are assigned to at least 3 genes. Data processing and visualization were performed in R (ver. 4.2) using in-house R scripts. GO, Gene Ontology; CC, cellular compartments; BP, biological process; MF, molecular function; log2FC, log2-fold change. The data used for generating the figure can be found in the **Supplementary Material**.

IAV H3N2/Fukui-infected cells induced the broadest GO term enrichment (73 significant terms) indicating extensive remodeling of the HLA-I ligandome. Enriched categories involved in stress response, translation, RNA metabolism, and antiviral defense (**Figures 4A**, left). Viral infection prominently enriched stress granule assembly (fold enrichment 12.16), mitotic spindle organization (fold enrichment 7.42), interferon-β production (fold enrichment 7.24) and antiviral defense pathways (fold enrichment 2.74) reflecting enhanced presentation of peptides linked to stress and innate immune activation.

Cellular compartments such as cytosol (fold enrichment 1.78), membrane (fold enrichment 2.33) nucleoplasm (fold enrichment 1.89), polysome (fold enrichment 7.64), catalytic step 2 spliceosome (fold enrichment 5.29), stress granule (fold enrichment 4.88), and kinetochore (fold enrichment 2.80) were enriched, consistent with reorganized translation and RNA processing machinery. RNA binding (fold enrichment 2.81), double-stranded RNA binding (fold enrichment 5.90), mRNA binding (fold enrichment 3.36), and ATPase activity (fold enrichment 2.86), ATP binding (fold enrichment 1.95), and cadherin binding (fold enrichment 3.55) dominated molecular function enrichment, illustrating robust antiviral ligandome remodeling (**Figure 4A**).

Zapnometinib treatment resulted in a more selective enrichment profiles (28 terms), largely confined to cell cycle regulation and RNA processing without antiviral pathway activation (**Figure 4B**). Enriched compartments included cell division (fold enrichment 4.70) mitotic cell cycle (fold enrichment 5.15), kinetochore (fold enrichment 4.32), microtubule organizing center (fold enrichment 5.65), midbody (fold enrichment 4.48), membrane (fold enrichment 2.57), cytosol (fold enrichment 1.79), and nucleoplasm (fold enrichment 1.92). RNA processing compartments including catalytic step 2 spliceosome (fold enrichment 6.77), spliceosome complex (fold enrichment 4.86), and nuclear speck (fold enrichment 2.62). RNA binding (fold enrichment 2.86), ATP binding (fold enrichment 1.86), and telomerase RNA binding (fold enrichment 15.94), with telomerase RNA binding showing the highest fold enrichment in this condition but no engagement of innate defense mechanisms, indicating that MEK inhibition selectively remodels the ligandome by suppressing proliferation-associated antigens without engaging antiviral defense mechanisms (**Figure 4B**).

Combined infection and zapnometinib treatment produced a distinct and attenuated enrichment pattern (17 terms) (**Figure 4C**). Biological process enrichment was minimal, dominated by adhesion-related cellular compartments such as adherens junction (fold enrichment 4.70), focal adhesion (fold enrichment 3.17), and intermediate filament cytoskeleton (fold enrichment 7.73), as well as enrichment in core cellular compartments including membrane (fold enrichment 2.50), cytosol (fold enrichment 1.76), and extracellular exosome (fold enrichment 2.26). RNA binding (fold enrichment 2.65) protein binding (fold enrichment 1.22) cadherin binding (fold enrichment 3.97) were enriched. RNA helicase activity (fold enrichment 7.23), ATPase activity (fold enrichment 3.17), and ATP binding (fold enrichment 1.85) demonstrated maintained catalytic activity enrichment in the combined condition. Classical antiviral categories observed during infection alone were suppressed, indicating that MEK inhibition dampens infection-driven antiviral stress responses and redirects antigen presentation toward adhesion-related pathways (**Figure 4C**) (**Supplementary Table 2** for top enriched pathways and complete lists).

#### 3.5.2 Gene-pathway network architecture: hub protein organization and functional integration

To complement the GO enrichment analysis, chord diagrams were generated to visualize the gene-pathway connections and identify key hub proteins driving functional changes under each condition. During IAV H3N2/Fukui infection, 271 genes formed a densely interconnected network (**Figure 4D**) that reveals the molecular drivers of the 73-term GO enrichment profile identified in the bubble plot (**Figure 4A**). Five hub proteins: IFIH1 (logFC 2.50, RIG-I-like receptor), DHX58 (logFC 5.77, RIG-I-like receptor), DDX3X (logFC 2.14, ATP-dependent RNA helicase), DDX21 (logFC 3.23, nucleolar RNA helicase), and HSP90AA1 (logFC −2.10, heat shock protein), were central to RNA sensing, helicase activity, and interferon signaling, reflecting the broad antiviral enrichment observed in the GO analysis. Upregulation of RIG-I-like receptor pathway components (DHX58 (logFC 5.77), IFIH1 (logFC 2.50)), and antiviral effectors (IFIT1 (logFC 9.48), IFI27 (logFC 8.04), OAS2 (logFC 5.14), OASL (logFC 5.15), eIF4G1 (logFC 8.97)) underscores a coordinated antiviral and translational remodeling response.

Zapnometinib treatment generated a smaller network of 110 genes with strong downregulation of cell cycle regulators (mitotic checkpoint kinase NEK2 (logFC -6.20), G2/M checkpoint kinase WEE1 (logFC -4.48), DNA replication licensing factor CDC6 (logFC -2.67), DNA polymerase epsilon POLE (logFC -5.21), topoisomerase II TOP2A (logFC -5.76), and chromosomal cohesion complex member SMC4 (logFC -6.67), and no antiviral hubs (**Figure 4E**). The sole upregulated hub, TEP1, linked telomerase RNA binding and ATP binding, suggesting selective activation of survival mechanisms during MEK inhibition. This confirms that zapnometinib suppresses proliferation-associated antigens while bypassing antiviral defense pathways.

The combined IAV H3N2/Fukui infection and zapnometinib treatment produced a reprogrammed network of 232 genes with distinct functional architecture, distinct from a mere overlay of the individual infection and treatment networks. RNA helicase family members (DDX17, MTREX, DHX34, DDX5, DDX47) were downregulated, indicating suppression of infection-driven antiviral helicase activity. Instead, adhesion pathways dominated driven by upregulation of CDH1 (E-cadherin, logFC 6.98) and SCYL1 (logFC 8.32), and concurrent downregulation of cytoskeletal components such as RDX (radixin, logFC -6.11), ATXN2L (ataxin 2-like, logFC -7.11), and ARHGAP1 (Rho GTPase-activating protein, logFC -5.42) (**Figure 4F**). This indicates a shift toward adhesion-related antigen presentation and reduced antiviral or cell cycle signals.

Overall, these analyses reveal three distinct ligandome phenotypes: (1) broad antiviral coordination during IAV H3N2/Fukui infection, (2) selective cell cycle suppression under zapnometinib treatment, and (3) adhesion-focused immune redirection under combined conditions. MEK inhibition thus acts as a selective ligandome modulator, redirecting immune recognition from antiviral stress responses toward cell-cell adhesion pathways during infection.

## 4 Discussion

Antiviral resistance in influenza viruses and the emergence of new variants remain major public health concerns. In this context, drug repurposing and therapeutic switching strategies are gaining increasing attention. Our findings demonstrate that zapnometinib possesses antiviral efficacy that can help to overcome limitations of currently licensed antivirals, either as monotherapy or in combination with standard-of-care drugs (15, 19, 26). Importantly, previous reports established a dual benefit of zapnometinib: direct antiviral activity and immunomodulatory effects, including suppression of pro-inflammatory cytokine and chemokine production that typically increase during infection (27, 28).

Mass spectrometry–based immunopeptidomics has previously been applied to identify the physiological targets of T-cell responses in cancer patients and to study how anticancer drugs affect the HLA ligandome (38, 55–57). Given that zapnometinib was initially developed as an anticancer drug, we used this approach to characterize its effects on the HLA-I ligandome of human lung adenocarcinoma cells during viral infection. MEK inhibitors are known to enhance HLA-I surface expression by blocking MAPK signaling, which negatively regulates HLA-I expression (33, 35, 58–60).

In contrast to other anticancer drugs (36, 37, 61), our quantitative analyses confirmed that zapnometinib treatment does not significantly alter HLA-I surface expression levels in Calu-3 cells. This observation is consistent with our flow cytometry results and supports the mechanistic insight that zapnometinib exerts its immunomodulatory effects predominantly through selective remodeling of the HLA-I ligandome composition rather than through altering bulk HLA-I expression levels. This mechanism contrasts with viral immune evasion strategies. Previous studies have shown that viral infections such as herpesviruses and HIV can interfere with antigen presentation by downregulating HLA-I surface expression (62–65). Notably, in our experiments, IAV H3N2/Fukui infection did not substantially reduce HLA-I surface expression in Calu-3 cells, suggesting that this respiratory virus strain does not employ HLA-I downregulation as a primary immune evasion strategy in epithelial cells. Calu-3 cells were specifically selected due to their physiological HLA allotype expression, robust IAV replication capacity, and established utility for quantitative immunopeptidomics. The stability of total HLA-I surface expression (**Figures 1B, C**) enabled sensitive detection of selective ligandome remodeling rather than global expression changes, revealing three mechanistically distinct HLA-I ligandome phenotypes (**Figures 4A–F**). Although IAV and IBV can modulate MHC-I profiles (53), these modulatory effects may be virus strain-specific and/or dependent on the host cell type, consistent with the tropism-dependent nature of influenza-host interactions.

LFQ analysis further revealed that zapnometinib treatment alters the relative abundance and composition of HLA-I–presented peptides. IAV-derived peptides represented only a minor presentation of the total HLA-I ligandome across all three conditions (IAV H3N2/Fukui infection, zapnometinib treatment, and combined), consistent with observations that viral peptides typically comprise a small proportion of the HLA-I immunopeptidome despite robust viral replication. Distinct modulation in HLA-I allotype distribution was observed upon zapnometinib treatment and IAV infection in Calu-3 cells, indicating that MEK inhibition affects peptide selection and presentation through allotype-selective mechanisms. Notably, only a subset of ligands (~3–14% per condition) passed our stringent significance criteria (log_2_ fold change ≥ 2 and adjusted p < 0.05), indicating that the observed effects are confined to defined fractions of the immunopeptidome rather than representing global shifts (**Figure 3**).While HLA-I ligands derive mainly from intracellular proteins, their abundance often correlates only weakly with source protein levels in the cellular proteome, reflecting the complex interplay of proteolytic processing, peptide generation kinetics, MHC binding affinity, TAP transporter selectivity, and intracellular subcellular localization that collectively determine which epitopes reach the cell surface (66–68). Thus, comprehensive interpretation requires integration with omics approaches and in silico prediction tools (69–72).

Our functional annotation analyses of HLA-I–presented source proteins revealed distinct remodeling of host antigen presentation in response to IAV infection and zapnometinib treatment. GO enrichment and network analyses provided systems-level view of how viral infection and MEK inhibition shape the HLA-I ligandome, emphasizing their differential impact on immune signaling and cellular function. IAV H3N2/Fukui infection induced a broad antiviral ligandome signature involving stress response, interferon signaling, RNA metabolism, and translation remodeling, consistent with the presentation of stress- and virus-associated antigens that promote CD8^+^ T cell activation. In contrast, zapnometinib treatment produced a narrower, proliferation-focused ligandome characterized by selective suppression of cell cycle–related proteins and RNA processing pathways without activating antiviral defenses. Combined infection and zapnometinib treatment resulted in a non-additive phenotype defined by enrichment of adhesion and communication pathways, suggesting a redirection of immune recognition from antiviral stress antigens toward adhesion-related epitopes.

Network analysis further highlighted how MEK inhibition selectively modulates the infection-driven antiviral network. eIF4G1 was strongly upregulated during IAV H3N2/Fukui infection but suppressed by zapnometinib treatment and further suppressed in combined infection and treatment condition. In contrast, IFIT1 remained substantially upregulated in combined treatment despite IAV H3N2/Fukui infection alone. IFI16, showed opposite behavior, downregulated in combined treatment despite infection-induced upregulation. These divergent interferon-stimulated gene (ISG) responses align with previous reports showing that MEK inhibition affects ISG expression and pro-inflammatory responses (27, 28), with clinical efficacy demonstrated in hospitalized COVID-19 patients (29). MAPK1 (ERK2) and MAPKAPK2 downregulation aligns with the mechanism of noncompetitive MEK inhibition, which attenuates ERK/MAPK signaling and consequently reduces the presentation of kinase-dependent peptides on HLA-I molecules, thereby reducing cellular abundance and/or immunological presentation of kinase-dependent proteins (19, 73, 74).

This study provides the first comprehensive characterization of HLA-I ligandome alterations following zapnometinib treatment using quantitative mass spectrometry in a human lung adenocarcinoma model. While overall HLA-I surface expression in Calu-3 cells remained unchanged, the composition and abundance of individual peptides within statistically defined subsets of the ligandome shifted in an allotype- and pathway-selective manner. A key limitation of this work is that all analyses were conducted exclusively in Calu-3 cells, a transformed epithelial cell line, which may not fully reflect the complexity of primary respiratory tissues *in vivo*. Future studies should therefore extend these findings to immunopeptidomic analyses of non-classical HLA molecules (HLA-E, -F, -G), the HLA-II ligandome, and primary respiratory epithelial cells to comprehensively assess the broader relevance of MEK-dependent immune remodeling. Comparative analyses with other MEK inhibitors or host-directed antivirals will further clarify whether the observed ligandome modulation represents a class-wide phenomenon or a mechanism specific to zapnometinib.

Together, these findings highlight the dual antiviral and immunomodulatory potential of zapnometinib, demonstrating that selective MEK inhibition reprograms antigen presentation during viral infection. By redirecting immune recognition from antiviral stress antigens toward adhesion-related epitopes, zapnometinib may alters the antigenic context available for CD8^+^ T cell surveillance in this cellular model. This ligandome-level remodeling provides additional mechanistic insight into host-directed antiviral therapy and supports further preclinical and clinical investigation of MEK inhibitors as candidate immunomodulatory antivirals.

## Data Availability

The data that support the findings of this study are available upon reasonable request from the corresponding author. The MS/MS data have been deposited to the ProteomeXchange consortium repository ( HYPERLINK "http://proteomecentral.proteomexchange.org)/"http://proteomecentral.proteomexchange.org) via the PRIDE partner repository [75] with the dataset identifier PXD041578.
